# The Adaption of Recent New Concepts in Neural Radiance Fields and Their Role for High-Fidelity Volume Reconstruction in Medical Images

**DOI:** 10.3390/s24185923

**Published:** 2024-09-12

**Authors:** Haill An, Jawad Khan, Suhyeon Kim, Junseo Choi, Younhyun Jung

**Affiliations:** School of Computing, Gachon University, Seongnam 13120, Republic of Korea; xenotic@gachon.ac.kr (H.A.); jkhanbk1@gachon.ac.kr (J.K.); kih629@gachon.ac.kr (S.K.); wnstl1234@gachon.ac.kr (J.C.)

**Keywords:** neural radiance field, medical image reconstruction, 2D–3D reconstruction

## Abstract

Volume reconstruction techniques are gaining increasing interest in medical domains due to their potential to learn complex 3D structural information from sparse 2D images. Recently, neural radiance fields (NeRF), which implicitly model continuous radiance fields based on multi-layer perceptrons to enable volume reconstruction of objects at arbitrary resolution, have gained traction in natural image volume reconstruction. However, the direct application of NeRF to medical volume reconstruction presents unique challenges due to differences in imaging principles, internal structure requirements, and boundary delineation. In this paper, we evaluate different NeRF techniques developed for natural images, including sampling strategies, feature encoding, and the use of complimentary features, by applying them to medical images. We evaluate three state-of-the-art NeRF techniques on four datasets of medical images of different complexity. Our goal is to identify the strengths, limitations, and future directions for integrating NeRF into the medical domain.

## 1. Introduction

Volume reconstruction using 2D images is attracting increasing interest in medical domains [1,2]. The primary rationale behind the burgeoning attention to this technology within medical domains lies in its capacity to learn intricate 3D structural information from a sparse collection of 2D images, thus allowing for providing a 2D medical image from a new view and voxel-level representation in 3D without additional physical image acquisition procedures. A potential 3D representation example is that of computed tomography (CT) imaging, which contains different regions of interest (ROI) within the human body. Volume reconstruction technologies would offer the display of the fine details of ROIs with their spatial relation in regard to neighboring organs in a non-invasive manner. In addition, the physical image acquisition step inherently exhibits the risk of motion artifacts. It is performed to obtain multiple scans by rotating around the human body, causing lengthy sessions that necessitate patient stillness. Volume reconstruction technologies would provide an increased chance of obtaining artifact-free 3D imaging. Another beneficiary can be 2D X-ray imaging. Volume reconstruction techniques are capable of synthesizing a new 2D X-ray image from views that are not included in the training stage. Volume reconstruction techniques would prevent additional physical image acquisition and reduce unnecessary radiation exposure.

A variety of deep learning concepts have been investigated to enable volume reconstruction using medical images. One of the primary streams has been convolutional neural networks (CNNs), renowned for their ability to extract local features from input data, which is particularly beneficial in capturing fine details within medical images. This enables CNNs to learn intricate patterns and relationships within data, contributing to accurate reconstructions [3,4,5]. However, a limitation of CNNs lies in their reliance on local features, which may result in limited context awareness, potentially leading to inaccuracies in reconstructing global anatomical structures [6]. To address such a limitation, researchers have turned to generative adversarial networks (GANs) [7]. GANs leverage a dual-network architecture comprising a generator and a discriminator. Through an adversarial learning process, wherein the generator aims to deceive the discriminator by generating increasingly realistic samples, GANs effectively learn the complex distribution of the training set and synthesize high-fidelity images [8]. One unignorable concern of GANs is the risk of mode collapse, where a generator produces images that merely replicate those present in the training set, leading to irrelevant reconstructions if new views are different from the training set [9].

Recently, there has been growing interest in neural radiance fields (NeRFs) [10] in computer graphics due to their groundbreaking capacity in view synthesis and volume reconstruction. NeRFs formulate an object as multi-layer perceptrons (MLPs) that can implicitly model a continuous radiance field. MLPs can be approximated with a collection of captured images and corresponding camera poses for an object. NeRFs have been demonstrated to maintain high fidelity in the geometry and appearance of an object. Moreover, with the learned continuous radiance field, the NeRF enables volume reconstruction of an object at arbitrary resolution and has the potential for super-resolution imaging. Because NeRF is a per-target process, it is unlikely to encounter the known issues in conventional deep learning technologies.

Despite its promise, the investigation of NeRFs in medical domains remains largely unexplored due to the fundamental difference between natural images in computer graphics and those in medical domains [11]. Unlike natural imaging, where the external geometry of an object, e.g., the surface, is captured through reflected lights, medical imaging involves penetrating the human body to reveal internal organs, tissues, and skeletons. This difference makes the direct application of NeRFs unfeasible. Moreover, the medical domains necessitate a high level of volume reconstruction. Precise delineation is paramount for medical tasks such as tumor localization and topological measurement.

In this study, we aim to introduce concepts of NeRFs developed for natural images and explore whether they could address the mentioned challenges when adapting to the medical domains. We determine three state-of-the-art NeRF techniques [12,13,14] that could be useful for the medical domains. They innovate the vanilla NeRF in either sampling strategy, feature encoding, or the use of complimentary features. We optimize all candidates to four medical image datasets by replacing the compositing formula with the X-ray radiation and the stratified fetching with a uniform manner. With our quantitative and qualitative results, we seek to identify the strengths, limitations, and future directions of integrating NeRF into the medical domain. The contributions of our work are summarized below:

- We introduce the emerging concept of NeRF, originally developed for natural images, into the medical domain to explore their potential applicability and utilities;
- We evaluate four state-of-the-art NeRF techniques by optimizing them to four distinct medical image datasets to identify their strengths and limitations;
- We conduct a comprehensive analysis with the traditional filtered back project (FDK) alternative [15] that is commonly employed for reconstructing medical images.

The rest of this paper is organized as follows. In Section 2, we review the other methods related to medical volume reconstruction and neural radiance field. In Section 3, we describe the comparative method in detail. We then explain our dataset, environment settings in Section 4, and evaluation metric, and follow this by presenting the evaluation results and providing detailed discussions in Section 5. Finally, our conclusions and directions for future work are explained in Section 6.

## 2. Related Works

### 2.1. Medical Volume Reconstruction

Volume reconstruction typically takes sample images, such as X-rays, as input data and attempts to learn the complex relationships between images and the corresponding reconstructed volume. Previous work in deep learning-based medical image reconstruction has mainly used CNNs to extract features from images and attempt 3D reconstruction. Henzler et al. [16] used a CNN architecture to reconstruct a 3D volume from multiple 2D X-ray images. In order to combine global and local features, the network was designed as an encoder–decoder structure that can be used at different resolutions. The encoder is tasked with transforming the abstraction of an image into an internal representation, encapsulating the information within the data. Conversely, the decoder is responsible for applying this representation to a specific instance, such as a volume. Shen et al. [17] proposed a deep neural network designed to learn the transformation from 2D projected radiographs to 3D anatomical details by incorporating convolutional modules within a high-dimensional feature space. This architecture facilitates the reconstruction of volumetric 3D CT images from limited views and utilizes an encoder–decoder structure for the 3D image reconstruction task, which is trained to effectively capture the relationship between feature space and image space. However, despite the ability of CNNs to extract a wide variety of features from medical images, there is still a concern that they may not fully understand the context of the 3D structure called a volume.

As an alternative, a GAN-based approach has been proposed that can provide complementary information between input data [18,19]. A GAN consists of two neural networks trained in parallel in a competitive manner: a generator and a discriminator. The generator network learns to generate realistic volumetric reconstructions from random noise or low-quality input data, while the discriminator network learns to distinguish between real volumes and those synthesized by the generator. Through this adversarial training process, the generator leverages feedback from the discriminator to gradually improve its ability to produce highly realistic volumetric reconstructions. Ying et al. [18] incorporated adversarial loss into the network architecture to facilitate learning of human anatomy from orthogonal views of X-rays. They also leveraged the generator network to augment the data dimensionality from 2D X-rays to 3D CT to enhance the reconstruction process. Ratul et al. [19] also proposed a novel approach by introducing class conditional networks. These networks utilize 2D feature maps of semantic segmentation as prior constraints to effectively guide volumetric reconstruction with improved accuracy and consistency. However, GANs remain at risk of generating structures that mimic those present in the training data rather than accurately estimating them from the input. As a result, the resulting volume structure can often result in reconstructions that are irrelevant to the input.

### 2.2. Neural Radiance Field

Since NeRFs [10] first appeared, researchers have made notable progress in enhancing implicit neural representations through a variety of improvements in encoding techniques, sampling strategies, feature integration, and more. For instance, Fourier feature mapping, as showcased in [20], has facilitated the learning of intricate functions by multi-layer perceptrons, thereby enabling the representation of complex objects or scenes with high-frequency details. Muller et al. [13] introduced a multi-resolution hash encoding method, significantly enhancing the efficiency of NeRF models by optimizing data structures and retrieval processes. Moreover, advancements in sampling strategy, such as the rendering approach employing conical frustums introduced by Barron et al. [12], have not only improved efficiency but also mitigated aliasing artifacts in multiscale training. Furthermore, Yu et al. [14] developed a method capable of accommodating multiple scenes by leveraging an image encoder to condition neural radiance fields on image features. Jain et al. [21] proposed an effective reconstruction technique with a small amount of data by applying the prior knowledge of a pre-trained image encoder trained on highly diverse 2D single-view image data.

However, these studies have focused on the field of natural images and are relatively less studied in the medical domain. Zha et al. [22] proposed a neural attenuation field to facilitate the reconstruction of 3D CT data, making it suitable for medical imaging. Building on the previous work, Fang et al. [23] incorporated a pre-trained denoising module to further improve data quality and reduce input projections. Nevertheless, due to the limited application of natural image-based NeRF techniques and the specificity of the medical domain, further research is needed. Therefore, in this paper, we aim to identify the strengths, limitations, and future directions of new techniques developed in a natural image-based NeRF when applied to the medical domain.

## 3. Comparative NeRF Methods and Optimization to Medical Domains

**Figure 1:**
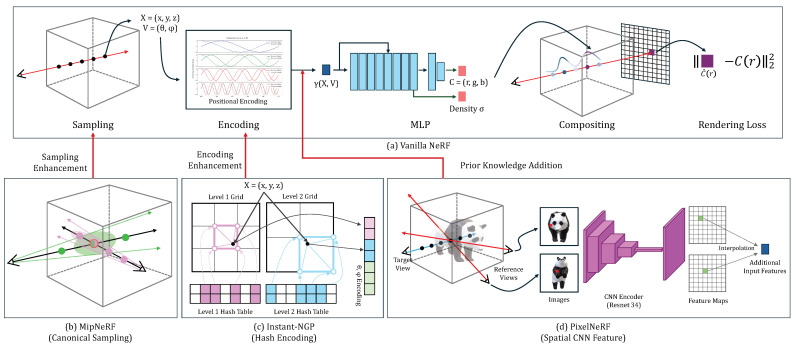
The architecture overview of the vanilla NeRF (**a**) and the three variations (**b**–**d**).

In this study, we aim to introduce the new concept of NeRFs developed for natural images and to explore their strengths, limitations, and future directions when adapting to the medical domains. We choose the vanilla NeRF as a baseline and introduce three state-of-the-art NeRF techniques [12,13,14] that could be useful for the medical domains. They innovate the vanilla NeRF in either sampling strategy, feature encoding, or the use of complementary features, as shown in Figure 1. We optimize all candidates to our four medical image datasets. The four sub-sections provide the details.

### 3.1. NeRF

The architecture overview of the vanilla NeRF and its three variations are shown in Figure 1. The NeRF learns the implicit function mapping 3D spatial coordinates X=(x,y,z) and viewing direction V=(θ,ϕ) to color C=(r,g,b) and density σ. The NeRF relies on the volume raycasting integral to reconstruct the geometry and appearance of an object using a set of captured images and corresponding camera poses for an object. The color and density of samples (voxels) obtained through the implicit function are accumulated into one pixel of an output image as follows:

(1) $$C\left(r\right)=\int_{t_{1}}^{t_{2}}T\left(t\right)\cdot \sigma \left(r\left(t\right)\right)\cdot c\left(r\left(t\right),d\right)\;dt,$$

$$\mathrm{where}\;\;T\left(t\right)=\exp\left(-\int_{t_{1}}^{t}\sigma \left(r\left(s\right)\right)\;ds\right)$$

where r(t) = o+td, *o* is the ray origin, *d* is the direction vector of the ray, and $t_{1}$ and $t_{2}$ are the near and far bounds in the ray. During training, NeRF minimizes the discrepancy between the synthesized pixel colors and the corresponding colors from the ground truth (captured) image. The loss function is based on the squared error between the color pairs as follows:

(2) $$L=\sum_{r\in R}{\left\|\hat{C}\left(r\right)-C\left(r\right)\right\|}_{2}^{2}$$

where *R* is the set of rays, $\hat{C}\left(r\right)$ is the synthesized colors, and C(r) is the ground truth. By iteratively optimizing the implicit function using the training data and minimizing the square error loss, NeRF learns the geometry and appearance of an object, which can be used to reconstruct its voxel-level representation in 3D and synthesize a 2D image from any new views. To adopt NeRF to medical images, we implement the attenuation field and uniform sampling.

### 3.2. Attenuation Field

Medical imaging involves X-rays penetrating the human body to capture internal structures, unlike natural imaging where the surface of an object is captured through reflected lights. This penetration is formulated as attenuation fields. The attenuation coefficient indicates the proportion of an X-ray absorbed when traversing various structures, contingent upon their densities. We map 3D coordinates X to the attenuation coefficients μ rather than dealing with color values in natural images. The X-ray source emits a cone-shaped beam with an initial intensity, which undergoes attenuation as the X-ray passes through diverse structures and culminates in the reception by a flat-panel detector. The attenuation field is typically formulated through the Beer–Lambert Law [24]:

(3) $$I=I_{0}e^{-\sum \mu_{i}\delta_{i}}$$

where *I* is the resultant attenuated intensity, $I_{0}$ is the initial intensity, μ is the attenuation coefficient, and δ denotes the sampling step.

### 3.3. Uniform Sampling

We rely on uniform sampling instead of selectively sampling points based on their importance. The stratified sampling is commonly used in natural images where certain regions (e.g., surface) are given high priority for 3D reconstruction of an object. However, this sampling is not ideal for the volume reconstruction of medical images to which every region equally contributes. We aim to cover the entire volume space and ensure that no regions of the volume are overlooked or over-represented by evenly distributing sampling points along a ray.

### 3.4. Three Variations of the Vanilla NeRF

We adopt three state-of-the-art NeRFs to facilitate the volume reconstruction of medical images by improving sampling strategy, feature encoding, and the use of complimentary features.

**MipNeRF (canonical sampling)**: MipNeRF [12] replaces the traditional point sampling with canonical region sampling, where rays are replaced with cones generated along the camera’s center (see Figure 1b). This approach allows for a more structured sampling strategy, capturing information from larger regions rather than individual points. By utilizing cones, MipNeRF can gather richer contextual information, leading to more robust and accurate 3D representations.

**Instant-NGP (hash encoding)**: Instant-NGP [13] introduces a novel feature encoding approach by utilizing multi-level hash to replace the conventional Fourier transform (see Figure 1c). Instant-NGP employs a learnable hash encoding mechanism to put encoding processes into training. This approach allows for a more flexible and adaptable feature encoding for the given task or dataset. This enhances the capacity to capture complex spatial information within an object and enables more effective 3D representation.

**PixelNeRF (spatial CNN feature)**: PixelNeRF [14] extends the capabilities of vanilla NeRF by leveraging additional CNN features extracted from images instead of solely relying on basic positional features (see Figure 1d). This fusion of image-derived features with geometric information not only enhances the ability to capture intricate details but also improves semantic consistency across diverse views.

## 4. Experiments

### 4.1. Dataset

We evaluated the applicability of four state-of-the-art NeRF techniques in the medical domain by utilizing four medical image datasets to represent diverse challenges encountered in medical volume reconstruction. Details of datasets are given in Table 1.

**Hard tissue reconstruction (Feet and Jaw)**: We consider the volume reconstruction of hard tissue (e.g., bones) to be similar to natural images. With the datasets, we explore how the new concepts for natural images can perform volume reconstruction in the medical domains. In addition, these datasets exhibit dense and irregular contour shapes. The Feet dataset [25] has a relatively thin structure, and the Jaw dataset [26] comprises the diverse shapes of teeth.

**Soft tissue reconstruction (Abdominal and Chest)**: We consider soft tissues to better represent the attenuation nature of medical images. Soft tissues such as muscles, organs, and vessels are frequently observed in medical images and it is critical to accurately reconstruct them. In addition, they tend to share similar densities and to be located in adjacency. It is important to distinguish and reconstruct them accurately. The abdominal dataset [27] contains multiple organs with similar densities. The chest dataset [28] captures the irregular structure of vessels inside the lungs and evaluates the ability to reconstruct fine-grained structures.

To train and validate NeRF techniques for medical volume reconstruction, we need paired datasets consisting of 2D images (e.g., X-ray) and their corresponding volumes (e.g., CT and MRI). However, collecting such paired datasets in a clinical setting is impractical because it is rare to obtain them at the same time. To overcome this acquisition issue, we use a technique called digital reconstruction radiography (DRR) to prepare 2D X-ray images from the collected CT volumes. Using the TIGRE tomography toolbox [29], we generated 50 X-ray images for each volume by covering 360° views.

### 4.2. Implementation

We implemented an evaluation standard to harmonize all four NeRF models (vanilla NeRF [10], MipNeRF [12], PixelNeRF [13], and Instant-NGP [14]) and the traditional FDK model [15] that is commonly employed for reconstructing medical images acquired by real-world scanners. We used a consistent image resolution of 512 × 512 to eliminate any bias that could arise from differences in image resolution and ensure that the quality of reconstruction could be compared directly. We also consistently scaled the input data (e.g., ray origins and directions) across all models since some models may process input differently due to their unique architectures (e.g., the inclusion of reference images in PixelNeRF). We applied a uniform sampling strategy. We ensured that the total number of sampled points across all rays was equivalent, thereby providing a fair comparison in terms of the volume covered by each model. In addition, MipNeRF utilized canonical sampling, where regions were sampled rather than individual points. We aligned the sampling density by increasing the number of samples in the other models. We experimented with all models using a TITAN RTX GPU with 24 GB VRAM.

All NeRF models are based on MLPs utilizing 128 channels per layer. Each of the NeRF models featured distinct MLP configurations: vanilla NeRF and MipNeRF comprised 8 layers with a skip connection concatenating input to the fifth layer; Instant-NGP consisted of 4 layers with skip connections to the third layer; and PixelNeRF included 3 layers with skip connections to the second layer. For positional and hash encoding, we adjusted the parameters defined in the original works [10,12,13] to better capture the characteristics of medical images. Our positional encoding used a higher frequency from 8 to 10 to enhance the representation of fine medical details. We used a hashmap size of 20 for our hash encoding to improve the granularity and precision of the encoded features. The training involved a batch size of 1024 rays, with each ray sampling points uniformly according to the width of volumes. For CNN feature extraction in PixelNeRF, we used three reference images per sampling point, utilized the resnet34 pre-trained model, and adjusted feature map sizes from 64 to 128 to enhance sensitivity to subtle medical (anatomical) structures. We utilized the Adam optimizer with a learning rate of $1\times 10^{-3}$ for Instant-NGP and $5\times 10^{-4}$ for the other architectures. Training persisted for 30K epochs, with early termination triggered if the evaluation metric deviated by less than 1% for three consecutive instances. We implemented the FDK model using the TIGRE tomography toolbox [29]. We suggest referring to the original works [10,12,13,14,15] for further details on implementation.

### 4.3. Evaluation Metric

We selected four evaluation metrics for volume reconstruction in medical images due to their complementary nature in assessing different aspects of image quality.

**Peak signal-to-noise ratio (PSNR)** measures the fidelity of the reconstructed volume by calculating the ratio of the maximum possible power of a signal to the power of corrupting noise. In the context of medical images, where accuracy and precision are crucial, PSNR helps in quantifying the level of noise or distortion present in the reconstructed volume.

**Structure similarity (SSIM)** evaluates the structural similarity between the original and reconstructed volumes by considering luminance, contrast, and structure. In medical images, preserving structural information such as boundaries and edges is essential. SSIM provides valuable insights into how well the reconstructed volume maintains these structural characteristics.

**Learned perceptual image patch similarity (LPIPS) [30]** measures the perceptual similarity between the original and reconstructed images by comparing deep features extracted from neural networks. LPIPS is particularly useful in evaluating how close the reconstructed volume is to the human perception of similarity, capturing nuances that may not be reflected in pixel-wise metrics like PSNR or SSIM.

**Gradient magnitude similarity deviation (GMSD) [31]** evaluates the deviation in gradient magnitude between the original and reconstructed images. GMSD is effective in evaluating the preservation of edges and fine details in the reconstructed volume, providing additional insights into the quality of the reconstruction, particularly in regions with high spatial variability.

## 5. Results and Discussions

The performance of the four NeRF techniques is quantitatively measured and compared with the traditional FDK technique, as shown in Table 2. It is observed that all NeRF techniques, except for MipNeRF, tend to outperform the traditional FDK technique for all medical datasets and evaluation metrics. Our results show that FDK struggles to capture the complex and fine details that NeRF techniques, particularly those with advanced encoding techniques (i.e., Instant-NGP and PixelNeRF), excel at. For example, Instant-NGP is substantially advantageous in preserving image structures by producing a large margin of 0.185 in 3D SSIM from the Feet dataset. Moreover, although FDK performs relatively well with simpler structures in hard tissues (e.g., Feet and Jaw datasets), its performance is substantially lower in datasets with complex soft tissues such as the Abdomen and Chest, which are more common in medical images, whereas NeRF techniques demonstrate a notable improvement. For instance, in the Abdominal dataset, Instant-NGP surpasses FDK by 8.3 in PSNR, reflecting its superior capability in handling the intricate details of soft tissues. In addition, Instant-NGP is much closer to the ground truth in terms of human visual perception by having a noticeable margin of 0.166 in LPIPS from the Chest dataset. Additionally, the GMSD for Instant-NGP is substantially lower ($3.7\times 10^{-6}$ vs. $3.3\times 10^{-5}$ in the Jaw dataset), indicating its superior ability to minimize gradient distortions and provide visually coherent reconstructions. As such, NeRF techniques achieve enhanced volume reconstruction in both image and human perception aspects, which suggests that this new concept could be an alternative to utilizing and enhancing the volume reconstruction of medical images.

We found that the performance of all NeRF techniques on soft tissue datasets tends to be lower than that on hard tissue datasets. For example, all NeRF techniques achieve a PSNR of 26.4 on average from the Abdominal dataset compared to 31.6 from the Feet dataset, and the most inferior difference was 7.4 PSNR from MipNeRF technique. This tendency may be due to the more complex but less-defined structures in soft tissues, making accurate reconstruction more challenging. The SSIM scores also reflect this trend by showing that structural similarity is consistently lower in soft tissues, e.g., with an average of 0.857 for the Abdomen soft tissue compared to 0.919 for the Feet hard tissue. This tendency is further supported by the LPIPS and GMSD metrics, where soft tissues exhibit higher values, suggesting the greater perceptual dissimilarities and gradient magnitudes compared to hard tissues. For instance, vanilla NeRF shows an LPIPS of 0.230 for the Abdominal soft tissue, substantially higher than the 0.099 recorded for the Feet hard tissue. These discrepancies highlight the inherent challenges of reconstructing soft tissues with existing NeRF techniques. In addition, it indicates that even with our NeRF modifications to adopt medical images, the existing NeRF techniques may still not be optimal for the volume reconstruction of medical images, such as soft tissues, and further research and investigation need to be conducted.

Among all NeRF techniques, Instant-NGP consistently outperforms across all medical image datasets and evaluation metrics. This suggests that hash-based learnable feature encoding of Instant-NGP can be the most valuable in reconstructing medical images. Compared to vanilla NeRF, where positional encoding is utilized, Instant-NGP resulted in a higher 3D SSIM of 0.943 in the Chest dataset compared to 0.885 from vanilla NeRF, along with substantially lower LPIPS (0.090 vs. 0.164) and GMSD ($3.9\times 10^{-6}$ vs. $2.7\times 10^{-5}$) values. PixelNeRF also outperforms vanilla NeRF, but the performance margins from PixelNeRF are no greater than those from Instant-NGP, e.g., 0.031 of PixelNeRF vs. 0.058 of Instance NGP in 3D SSIM in Chest dataset. The results may suggest that the use of additional CNN features (i.e., PixelNeRF) can be useful but is not the most effective in medical images. MipNeRF consistently shows the lowest performances across all experiments. For example, in the Abdominal dataset, MipNeRF shows substantially lower PSNR (17.3) and SSIM (0.709) compared to other models, along with higher LPIPS (0.512) and GMSD ($1.7\times 10^{-3}$). It suggests that the introduction of regional (canonical) sampling, which is a core feature of MipNeRF, may be detrimental in the context of medical images.

**Figure 2:**
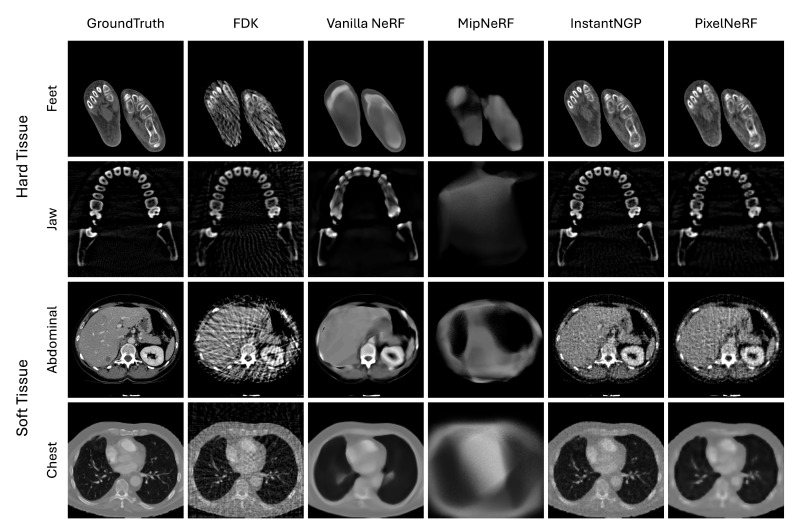
Visual comparison of four NeRF variations—vanilla NeRF, MipNeRF, Instant-NGP, and PixelNeRF—to the traditional FDK using four medical image slices (each row).

In Figure 2, we show the visual comparison of volume reconstruction results from four NeRF techniques and the traditional FDK technique using four medical image slices. Consistent with the numerical evaluation metrics, all NeRF techniques, except for MipNeRF, tend to surpass the FDK technique in both hard and soft tissue datasets. The dense and well-defined structures in the hard tissue datasets pose a critical challenge for the FDK method, where it leads to distinctive streaking artifacts, particularly evident in the reconstructions of important high-frequency details—e.g., the boundary of structures—that obscure useful details. NeRF techniques, particularly Instant-NGP and PixelNeRF, handle these dense structures more effectively by preserving the sharpness and clarity of the bone contours. On the other hand, the soft tissue datasets present a different challenge with their complex, less-defined structures and varying tissue densities. Here, FDK struggles even more, resulting in noisy reconstructions that fail to capture the subtle gradations within the tissues. In contrast, NeRF techniques, especially Instant-NGP, show a superior ability to reconstruct these intricate details, offering clearer and more detailed images of soft tissue structures. Note that the level of detail and accuracy varies among four different NeRF techniques.

We observe that the NeRF techniques tend to be, to some extent, sensitive to the type of tissue being reconstructed. Hard tissues typically present more defined edges and higher contrast compared to soft tissues, which makes them easier to reconstruct accurately using NeRF techniques. The clarity and detail in the feet and jaw regions reflect this, as the NeRF techniques, especially Instant-NGP and PixelNeRF, successfully capture the complex structures of bones with minimal artifacts. This enhancement can be attributed to the ability of NeRF techniques to handle high-frequency details and sharp edges. On the other hand, soft tissues tend to have lower contrast and more homogeneous textures, which present greater challenges for accurate reconstruction. NeRF techniques, while still outperforming the traditional FDK technique, show varying degrees of enhancement in these regions. The smoother transitions and less-defined boundaries in soft tissues lead to reconstructions that, while artifact-free, sometimes appear less detailed or slightly blurred. This outcome suggests that while NeRF techniques are effective at reducing noise and artifacts, additional refinement and optimization may be necessary to enhance their ability to better capture the subtle details present in soft tissue structures.

Among all NeRF techniques, Instant-NGP resulted in the greatest similarity to ground truth on all medical images. Medical images vary in objects to be reconstructed. The capability of Instant-NGP to learn a feature representation optimized for dataset-specific characteristics can address this variation, thus being able to capture the complex spatial relationships within medical images and to produce high-fidelity reconstructions. We also observe that the use of additional CNN features (i.e., PixelNeRF) helps enhance the reconstruction of internal small structures (see the teeth in Jaw dataset by comparing PixelNeRF with vanilla NeRF).

PixelNeRF and vanilla NeRF produce less-pronounced results than Instant-NGP. This is due to the reliance on frequency-based encoding. Frequency encoding decomposes the input image into high-frequency and low-frequency components and then uses the low frequency to provide the shape of the image and the high frequency to provide detailed texture. Training is performed preferentially on the low-frequency components, and they tend to prioritize capturing the overall shape and structure of the image rather than focusing on fine-grained details and textures. In contrast, the use of multi-level hash encoding in Instant-NGP facilitates both the overall shape and local textures.

MipNeRF shows the worst visual performance. This is likely due to its regional (canonical) sampling, which theoretically facilitates a more robust feature representation over image noises than conventional discrete point sampling but performs poorly when adapted to medical images. It seems that the regional sampling makes each point smooth and can have an adverse effect on capturing the fine detail and complex spatial relationships that characterize medical images.

In Table 3, we present a comparative analysis of rendering (inference) times across the four NeRF techniques alongside the traditional FDK technique using four distinct medical image datasets. Although the FDK method consistently shows the shortest inference times across all datasets, the corresponding image reconstruction results highlight a critical trade-off between speed and quality. While Instant-NGP and PixelNeRF require more processing time (e.g., 1.016 s and 2.189 s for the Feet dataset, respectively), our visual reconstruction results show that they achieve substantially higher reconstruction quality than FDK, particularly in preserving fine details and minimizing artifacts. It is observed that FDK is consistent between the two different types of datasets (i.e., hard vs. soft Tissues) but NeRF techniques result in higher variations, e.g., 8.546 in the Feet and Abdominal from vanilla NeRF. This tendency indicates that the existence of dense structures (i.e., soft tissues) requires more sampling for NeRF techniques to achieve accurate volume reconstruction.

The computational performance difference among the four NeRF techniques can be attributed to several factors. Instant-NGP benefits from the parallel low-level processing power of CUDA implementation by showing much lower computation times compared to other NeRF techniques, e.g., 2.591 of Instant-NGP vs. 5.493 of PixelNeRF in the Chest dataset. MipNeRF requires sampling the larger regions (i.e., canonical sampling) compared to the vanilla NeRF, and PixelNeRF performs the additional computation of CNN features, but their computational costs are marginally increased.

## 6. Conclusions and Future Works

In this work, we propose a comparative analysis of NeRF techniques, which are originally developed for natural images, against the traditional FDK technique for medical image reconstruction. We optimize the state-of-the-art NeRF techniques to better adapt to medical images and conduct quantitative and qualitative evaluations using distinct medical image datasets and evaluation metrics. Our results highlight that NeRF techniques offer noticeable enhancements in terms of reconstruction quality and detail preservation; however, as a trade-off, a longer computation time is required when compared to FDK techniques. Our analysis suggests that the visual gains from NeRF techniques would compromise the accompanying computational complexity, and this new concept of NeRF could be an alternative to utilizing and enhancing the volume reconstruction of medical images. Our experiments also indicate that Instant-NGP is a preferred solution due to its capability to capture complex anatomical details with higher accuracy and faster computation times.

Our results identify several future works to make them viable for medical applications and enhance the scalability and practicality of NeRF in real-world settings. Among the challenges identified, one of the most critical is insufficient evidence to support the feasibility of NeRF techniques in real clinical situations. Our experiments relied on medical image datasets that paired 3D CT with 2D simulated images using the DRR technique. While the DRR technique has been widely used in several 3D image deep learning domains [18,32,33]—e.g., generative adversarial network (GAN) or transformer—to simulate the 2D–3D paired environments, we note that it may be limited in fully representing real clinical situations and, thus, a more thorough evaluation using real-world clinical datasets has to be conducted to further investigate the clinical feasibility of NeRF techniques. We are now continuing to try to acquire new, real-world pair datasets from our medical institutions, after which we plan to identify new, valuable, in-depth findings on NeRF techniques.

We also note that current state-of-the-art NeRF techniques are sufficient in accurately reconstructing hard tissues but there is still room for improvement in the reconstruction of soft tissues that are more common in medical images (see Table 2 and Figure 2). Our future work could explore leveraging the embedding of generalized prior knowledge. We note that NeRF techniques are inherently designed to highly fine-tune the target patient and lack general features that are exhibited in large populations. Generalized prior knowledge can be introduced by pre-training NeRF using features from a diverse set of patients, which could then be fine-tuned using a small dataset of 2D images specific to the target patient. This approach could improve the accuracy of the reconstruction of soft tissue by utilizing both general and patient-specific features. Similarly, we also could leverage pre-operative volumes to facilitate the volume reconstruction of post-operative 2D images by performing coarse reconstruction using the pre-operative data and fine-tuning with spare post-operative 2D images. Another challenge is that NeRF techniques, due to training on a specific scene, cannot be easily generalized to other scenes without incurring the high cost of re-training. Our new strategy to leverage the generalized prior knowledge could be effective in addressing the re-training cost. It enables faster online fine-tuning by focusing on relevant features rather than learning an entire new scene from scratch.

## Figures and Tables

**Table 1:** Details of four experimental datasets.

Type	Dataset	Volume Size	Voxel Size (mm)	Number of X-ray Images	Viewing Range
Hard Tissue	Feet	512 × 512 × 250	0.4023 × 0.4023 × 0.5	50	0–360°
	Jaw	256 × 256 × 256	1.0 × 1.0 × 1.0	50	0–360°
Soft Tissue	Abdominal	512 × 512 × 129	0.57 × 0.57 × 1.6	50	0–360°
	Chest	128 × 128 × 128	1.0 × 1.0 × 1.0	50	0–360°

**Table 2:** Quantitative volume reconstruction comparison of the four NeRF variations—vanilla NeRF, MipNeRF, Instant-NGP, and PixelNeRF—to the traditional FDK using four medical image datasets based on four metrics. The best score is in blue and the worst is in red.

Type	Dataset	Models	3D PSNR↑	3D SSIM↑	LPIPS↓	GMSD↓
Hard Tissue	Feet	FDK	28.9	0.790	0.382	$1\times 10^{-4}$
		Vanilla NeRF	28.9	0.905	0.099	$7.8\times 10^{-5}$
		MipNeRF	24.5	0.832	0.220	$4\times 10^{-4}$
		Instant-NGP	37.6	0.975	0.037	$1.2\times 10^{-5}$
		PixelNeRF	35.4	0.965	0.047	$1.9\times 10^{-5}$
	Jaw	FDK	30.3	0.857	0.266	$3.3\times 10^{-5}$
		Vanilla NeRF	30.1	0.872	0.351	$4.2\times 10^{-5}$
		MipNeRF	22.5	0.609	0.690	$6\times 10^{-4}$
		Instant-NGP	34.4	0.941	0.143	$3.7\times 10^{-6}$
		PixelNeRF	31.9	0.913	0.303	$2\times 10^{-5}$
Soft Tissue	Abdominal	FDK	24.0	0.735	0.441	$2\times 10^{-4}$
		Vanilla NeRF	26.6	0.891	0.230	$1\times 10^{-4}$
		MipNeRF	17.3	0.709	0.512	$1\times 10^{-3}$
		Instant-NGP	32.3	0.942	0.183	$1.5\times 10^{-5}$
		PixelNeRF	29.2	0.888	0.237	$4\times 10^{-5}$
	Chest	FDK	25.8	0.744	0.256	$4.4\times 10^{-5}$
		Vanilla NeRF	27.9	0.885	0.164	$2.7\times 10^{-5}$
		MipNeRF	15.1	0.497	0.543	$1.2\times 10^{-3}$
		Instant-NGP	31.6	0.943	0.090	$3.9\times 10^{-6}$
		PixelNeRF	28.7	0.912	0.121	$1.6\times 10^{-5}$

**Table 3:** Rendering (inference) time comparison of the four NeRF variations—vanilla NeRF, MipNeRF, Instant-NGP, and PixelNeRF—to the traditional FDK using four medical image datasets. The best score is in blue and the worst is in red.

Model	Hard Tissue (sec)		Soft Tissue (sec)	
	Feet	Jaw	Abdominal	Chest
FDK	0.652	1.284	1.911	0.872
Vanilla NeRF	2.183	9.023	10.729	5.103
MipNeRF	2.748	10.162	11.285	5.394
Instant-NGP	1.016	4.201	4.978	2.591
PixelNeRF	2.189	9.657	10.878	5.493

## Data Availability

Data are contained within the article.

## References

[1] Häggström I., Schmidtlein C.R., Campanella G., Fuchs T.J. (2019). DeepPET: A deep encoder–decoder network for directly solving the PET image reconstruction inverse problem. Med. Image Anal..

[2] Zeng G.L. (2010). Medical Image Reconstruction.

[3] Guo Y., Cai J., Jiang B., Zheng J. (2018). Cnn-based real-time dense face reconstruction with inverse-rendered photo-realistic face images. IEEE Trans. Pattern Anal. Mach. Intell..

[4] Tan C., Lv S., Dong F., Takei M. (2018). Image reconstruction based on convolutional neural network for electrical resistance tomography. IEEE Sens. J..

[5] Ravishankar S., Ye J.C., Fessler J.A. (2019). Image reconstruction: From sparsity to data-adaptive methods and machine learning. Proc. IEEE.

[6] Minnema J., Wolff J., Koivisto J., Lucka F., Batenburg K.J., Forouzanfar T., van Eijnatten M. (2021). Comparison of convolutional neural network training strategies for cone-beam CT image segmentation. Comput. Methods Programs Biomed..

[7] Goodfellow I., Pouget-Abadie J., Mirza M., Xu B., Warde-Farley D., Ozair S., Courville A., Bengio Y. (2020). Generative adversarial networks. Commun. ACM.

[8] Creswell A., White T., Dumoulin V., Arulkumaran K., Sengupta B., Bharath A.A. (2018). Generative adversarial networks: An overview. IEEE Signal Process. Mag..

[9] Ferreira A., Li J., Pomykala K.L., Kleesiek J., Alves V., Egger J. (2024). GAN-based generation of realistic 3D volumetric data: A systematic review and taxonomy. Med. Image Anal..

[10] Mildenhall B., Srinivasan P.P., Tancik M., Barron J.T., Ramamoorthi R., Ng R. (2021). Nerf: Representing scenes as neural radiance fields for view synthesis. Commun. ACM.

[11] Molaei A., Aminimehr A., Tavakoli A., Kazerouni A., Azad B., Azad R., Merhof D. Implicit neural representation in medical imaging: A comparative survey. Proceedings of the IEEE/CVF International Conference on Computer Vision.

[12] Barron J.T., Mildenhall B., Tancik M., Hedman P., Martin-Brualla R., Srinivasan P.P. Mip-nerf: A multiscale representation for anti-aliasing neural radiance fields. Proceedings of the IEEE/CVF International Conference on Computer Vision.

[13] Müller T., Evans A., Schied C., Keller A. (2022). Instant neural graphics primitives with a multiresolution hash encoding. ACM Trans. Graph. (Tog).

[14] Yu A., Ye V., Tancik M., Kanazawa A. pixelnerf: Neural radiance fields from one or few images. Proceedings of the IEEE/CVF Conference on Computer Vision and Pattern Recognition.

[15] Feldkamp L.A., Davis L.C., Kress J.W. (1984). Practical cone-beam algorithm. J. Opt. Soc. Am. A.

[16] Henzler P., Rasche V., Ropinski T., Ritschel T. (2018). Single-image tomography: 3D volumes from 2D cranial X-rays. Proceedings of the Computer Graphics Forum.

[17] Zhu B., Liu J.Z., Cauley S.F., Rosen B.R., Rosen M.S. (2018). Image reconstruction by domain-transform manifold learning. Nature.

[18] Ying X., Guo H., Ma K., Wu J., Weng Z., Zheng Y. X2CT-GAN: Reconstructing CT from biplanar X-rays with generative adversarial networks. Proceedings of the IEEE/CVF Conference on Computer Vision and Pattern Recognition.

[19] Ratul M.A.R., Yuan K., Lee W. CCX-rayNet: A class conditioned convolutional neural network for biplanar X-rays to CT volume. Proceedings of the 2021 IEEE 18th International Symposium on Biomedical Imaging (ISBI), IEEE.

[20] Tancik M., Srinivasan P., Mildenhall B., Fridovich-Keil S., Raghavan N., Singhal U., Ramamoorthi R., Barron J., Ng R. (2020). Fourier features let networks learn high frequency functions in low dimensional domains. Adv. Neural Inf. Process. Syst..

[21] Jain A., Tancik M., Abbeel P. Putting nerf on a diet: Semantically consistent few-shot view synthesis. Proceedings of the IEEE/CVF International Conference on Computer Vision.

[22] Zha R., Zhang Y., Li H. (2022). NAF: Neural attenuation fields for sparse-view CBCT reconstruction. Proceedings of the International Conference on Medical Image Computing and Computer-Assisted Intervention.

[23] Fang Y., Mei L., Li C., Liu Y., Wang W., Cui Z., Shen D. (2022). Snaf: Sparse-view cbct reconstruction with neural attenuation fields. arXiv.

[24] Swinehart D.F. (1962). The beer-lambert law. J. Chem. Educ..

[25] Osirix-DICOM Image Library. https://www.osirix-viewer.com/resources/dicom-image-library/.

[26] Open Scientific Visualization Datasets. https://klacansky.com/open-scivis-datasets/.

[27] Soler L., Hostettler A., Agnus V., Charnoz A., Fasquel J.B., Moreau J., Osswald A.B., Bouhadjar M., Marescaux J. (2010). 3D Image Reconstruction for Comparison of Algorithm Database. https://www.ircad.fr/research/data-sets/liver-segmentation-3d-ircadb-01.

[28] Armato III S.G., McLennan G., Bidaut L., McNitt-Gray M.F., Meyer C.R., Reeves A.P., Zhao B., Aberle D.R., Henschke C.I., Hoffman E.A. (2011). The lung image database consortium (LIDC) and image database resource initiative (IDRI): A completed reference database of lung nodules on CT scans. Med. Phys..

[29] Biguri A., Dosanjh M., Hancock S., Soleimani M. (2016). TIGRE: A MATLAB-GPU toolbox for CBCT image reconstruction. Biomed. Phys. Eng. Express.

[30] Zhang R., Isola P., Efros A.A., Shechtman E., Wang O. The unreasonable effectiveness of deep features as a perceptual metric. Proceedings of the IEEE Conference on Computer Vision and Pattern Recognition.

[31] Xue W., Zhang L., Mou X., Bovik A.C. (2013). Gradient magnitude similarity deviation: A highly efficient perceptual image quality index. IEEE Trans. Image Process..

[32] Aubert B., Cresson T., de Guise J.A., Vazquez C. (2022). X-ray to DRR images translation for efficient multiple objects similarity measures in deformable model 3D/2D registration. IEEE Trans. Med. Imaging.

[33] Geng H., Fan J., Yang S., Chen S., Xiao D., Ai D., Fu T., Song H., Yuan K., Duan F. (2024). DSC-Recon: Dual-Stage Complementary 4D Organ Reconstruction from X-ray Image Sequence for Intraoperative Fusion. IEEE Trans. Med. Imaging.

